# Evolution of the surface atomic structure of multielement oxide films: curse or blessing?†

**DOI:** 10.1039/d3na00847a

**Published:** 2023-11-02

**Authors:** Giada Franceschi, Renè Heller, Michael Schmid, Ulrike Diebold, Michele Riva

**Affiliations:** a Institute of Applied Physics, TU Wien Wiedner Hauptstraβe 8-10/E134 1040 Vienna Austria franceschi@iap.tuwien.ac.at; b Institute of Ion Beam Physics and Materials Research Helmholtz-Zentrum Dresden-Rossendorf e.V., Bautzner Landstraße 400 01328 Dresden Germany

## Abstract

Atomically resolved scanning tunneling microscopy (STM) and X-ray photoelectron spectroscopy (XPS) are used to gain atomic-scale insights into the heteroepitaxy of lanthanum–strontium manganite (LSMO, La_1−*x*_Sr_*x*_MnO_3−*δ*_, *x* ≈ 0.2) on SrTiO_3_(110). LSMO is a perovskite oxide characterized by several composition-dependent surface reconstructions. The flexibility of the surface allows it to incorporate nonstoichiometries during growth, which causes the structure of the surface to evolve accordingly. This happens up to a critical point, where phase separation occurs, clusters rich in the excess cations form at the surface, and films show a rough morphology. To limit the nonstoichiometry introduced by non-optimal growth conditions, it proves useful to monitor the changes in surface atomic structures as a function of the PLD parameters and tune the latter accordingly.

## Abstract 2

Tracking the alterations in atomic-level surface structures of complex oxide films is a practical approach for achieving ideal film stoichiometries and surface morphologies.

## Graphical abstract

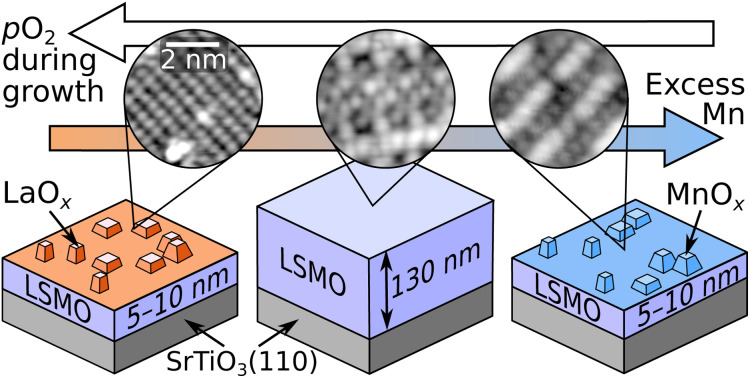

## 1. Introduction

Perovskite oxides dominate a host of established and emerging technologies due to their extraordinary tunability.^1–4^ Characterized by the chemical formula ABO_3_, perovskites and related crystal structures can accommodate about 30 elements on the A site and over half the periodic table on the B site.^5^ This opens up the attractive possibility to control the interplay between spin, charge, orbital, and lattice degrees of freedom and achieve unique properties.^3^ One example is La_1−*x*_Sr_*x*_MnO_3−*δ*_ (lanthanum–strontium manganite, LSMO), which shows doping-dependent transitions from metal to insulator and from (anti)ferro- to paramagnetic, as well as interesting catalytic properties.^6–10^

Because of their high sensitivity to stoichiometry and crystal structure changes,^11^ the properties of perovskite oxides are best explored by working with single-crystalline samples or bulk-like epitaxial thin films. The growth of ideal perovskite-oxide films is challenging, however. In pulsed laser deposition (PLD) – the technique of choice for multielement oxides – many parameters can affect preferentially one element or another in the compound. These parameters include the laser energy density, spot size, pulse duration, deposition geometry, pressure and nature of the ambient gas, and deposition rate.^12–15^ Depending on the precise values of the deposition parameters (sometimes hard to reproduce in different laboratories^13,16,17^), elements may be preferentially ablated at the target, preferentially scattered by the background gas, and have preferential sticking to the substrate.^13^ Put simply, stoichiometric targets do not warrant stoichiometric films. The crystallinity, morphology, and other macroscopic properties are also influenced. The effects are more pronounced at thicknesses larger than a few nanometres. LSMO films exemplify the struggle. Their morphology, composition, transport, and magnetic properties are highly sensitive to the growth conditions.^16,18–24^ Crystalline precipitates during nonstoichiometric growth are common.^25,26^

The causes for morphological roughening are numerous and intertwined. In the simple case of one-component films, deposition rate, energetics, attachment kinetics at step edges, mechanical stress, angle dependent rate, capillarity, viscous flow, and nucleation are known to be relevant.^27–32^ These effects are expected to play a role also within the growth of perovskite oxides. This work demonstrates that additional effects – not considered in traditional models and related to the surface atomic details of the growing films – are important for understanding and controlling the complex growth behaviours of perovskite oxides.

Perovskite oxides are known to exhibit a host of composition-dependent atomic-scale surface structures (also named surface reconstructions).^33–40^ Previous studies on SrTiO_3_(110) have already shown that these reconstructions are critically important during epitaxial film growth. If the deposition parameters are not optimized and the growth is nonstoichiometric, excess cations can segregate to the film surface^39^ and alter its atomic structure. The nonstoichiometry of the deposited material might be minute but its influence on the surface atomic structure can be detected by reflection high-energy electron diffraction (RHEED),^41^ low-energy electron diffraction (LEED), and scanning tunneling microscopy (STM).^39^ Importantly, these changes can alter the growth mechanisms^42,43^ and the surface morphology: if reconstructions with different sticking properties develop and coexist on the surface, pits might develop on the low-sticking areas.^13^

The existence of surface reconstructions does not solely bring negative implications. As it was shown for SrTiO_3_,^39^ it also opens up attractive opportunities. In cases where all the introduced non-stoichiometry segregates to the surface of the growing film, optimal growth conditions and stoichiometric growth with virtually unlimited accuracy can be achieved by monitoring the changes in the surface atomic structure as a function of film thickness and deposition parameters.^39^

This work builds on previous studies on SrTiO_3_ homoepitaxy. It focuses on LSMO films deposited on well-defined SrTiO_3_(110) substrates (see Section S1 of the ESI† for details on the setups and the growth) ref. 58. It investigates how the surface atomic structures of LSMO evolve during growth as a function of different parameters and film thicknesses, and how to leverage such changes to optimize the deposition parameters. The concepts are showcased by depositing under controlled conditions, where one parameter at a time is carefully modified – in this case, the value of the O_2_ background pressure (*p*_O_2__) within the incongruent transfer regime, where the lighter Mn species scatter more than the heavier La and Sr, leading to enrichment in La and Sr (Mn) at higher (lower) O_2_ pressures. The surface evolution is monitored with XPS and STM. Akin to SrTiO_3_, non-stoichiometries shift the surface atomic structure along established surface phase diagrams, following their substantial segregation to the surface.^40,44^ When the excess material cannot be accommodated by a suitable surface reconstruction, it precipitates in the form of clusters rich in the excess cations. Nonetheless, appropriate ultrahigh vacuum (UHV)-based surface treatments can heal the surface. A method is presented to optimize the PLD parameters to grow high-quality multielement films. Different from typical practices, it does not rely on *ex-situ*, *post-mortem*, bulk analyses. Rather, it leverages the changes in surface atomic structure that follow the partial segregation of non-stoichiometry. *Ex-situ* bulk analyses are offered to support the quality of the films and provide a link with standard characterization techniques.

## 2. Insights into the growth of LSMO(110)

**Fig. 1:**
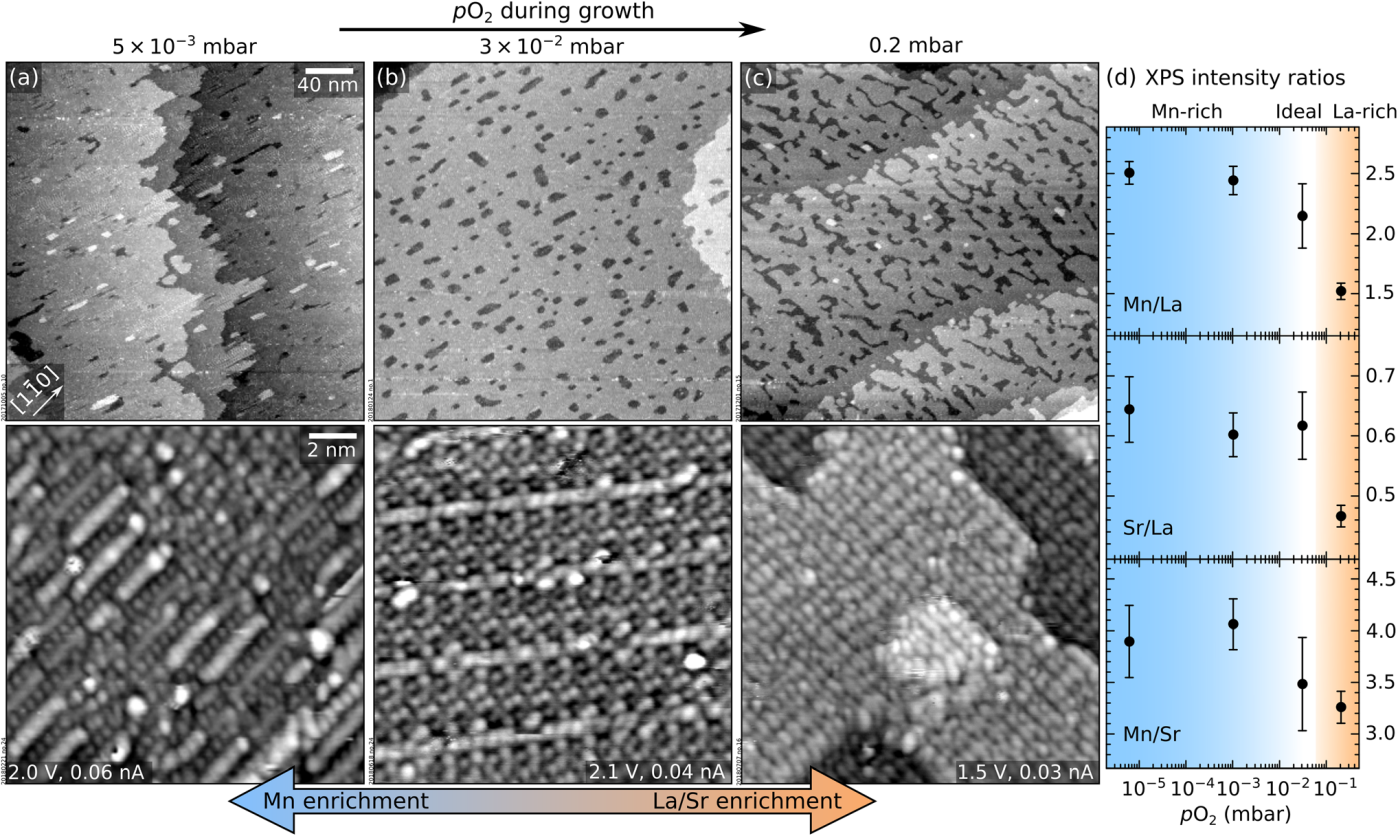
Effect of *p*_O_2__ during PLD on the morphology and composition of LSMO(110) films. (a–c) Top row: 300 × 300 nm^2^ STM images (*V*_sample_ = +2 V, *I*_tunnel_ = 0.2 nA) of thin films (9 layers, ≈ 2.5 nm thickness) grown at 1 Hz, 1.9 J cm^−2^, 700 °C, and 5 × 10^−3^ mbar ≤ *p*_O_2__ ≤ 0.2 mbar; bottom row: 15 × 15 nm^2^ STM images, high-pass-filtered for displaying purposes. (d) XPS intensity ratios of Mn 2p, La 4d, and Sr 3d peaks as a function of *p*_O_2__; lower *p*_O_2__ yields Mn-richer surfaces (the analysis includes an additional datapoint of a 9-layer-thick film grown at 5 × 10^−6^ mbar). Note that the Sr signal originating from the substrate cannot be decoupled from the one in the film.

This section addresses the role of *p*_O_2__ during PLD on the morphology and composition of LSMO(110) films. Fig. 1a–c show the STM morphology of three thin films grown at *p*_O_2__ values ranging between 5 × 10^−3^ mbar and 0.2 mbar (all other parameters are nominally the same: 1 Hz laser repetition rate, 1.9 J cm^−2^ laser fluence, 700 °C substrate temperature). All films have the same thickness of ≈2.5 nm or 9 layers, where one layer corresponds to the separation between two (110) planes, ≈0.276 nm.

In Fig. 1, all films appear atomically flat on the scale of a few hundred nanometres (top row). On a smaller scale (bottom row), different surface structures with different periodicities (*i.e.*, different surface reconstructions) are evident. The same reconstructions were observed by depositing sub-monolayer amounts of Mn on a stoichiometric film in order to establish a quantitative surface phase diagram of LSMO(110).^44^ Comparing this phase diagram with Fig. 1 reveals that the films grown at lower pressures exhibit reconstructions richer in Mn. This is confirmed by the XPS analysis of Fig. 1d. Note that the variation of the Mn/La XPS intensity ratio (Fig. 1d) by more than a factor of 1.6 is much higher than what would be possible in perovskite-type LSMO according to the bulk phase diagram.^45^ While STM shows different surface reconstructions (Fig. 1a–c), all these surface phases are based on the same perovskite lattice of the underlying layers.^44^ There is no evidence for the formation of different phases in the deeper layers of these films. This indicates that the stoichiometry variations at the surface are larger than in the deeper layers of the film.

**Fig. 2:**
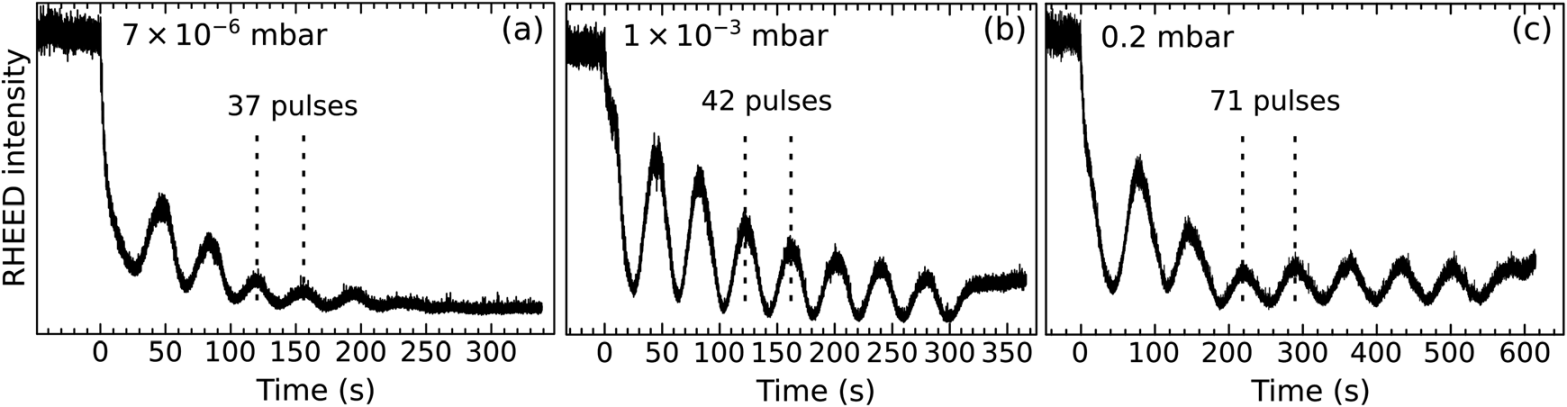
RHEED oscillations during the growth of thin LSMO(110) films (≈2.5 nm thick) at different *p*_O_2__ (values indicated in the respective panels) and otherwise identical growth parameters (1 Hz, 1.9 J cm^−2^, 700 °C). Note that the data do not correspond to the same films as shown in Fig. 1.

Fig. 2 displays the intensity of the specular RHEED spot *versus* the deposition time for three *p*_O_2__ values. All depositions occur in a layer-by-layer mode (one RHEED oscillation equals one layer). Higher pressures produce longer periods, an indication that less material reaches the substrate. Moreover, the intensities of the minima and maxima differ for the three films. This is not necessarily always an indication for better or worse layer-by-layer growth, however. The different surface reconstructions developing during growth (Fig. 1) could potentially cause diffraction conditions different from the starting point, which may explain some amplitude variations of the intensity oscillations.^13^ Nevertheless, the more pronounced decay observed in Fig. 2a probably indicates the slightly rougher surface morphology of the corresponding film (observed in STM, not shown here).

**Fig. 3:**
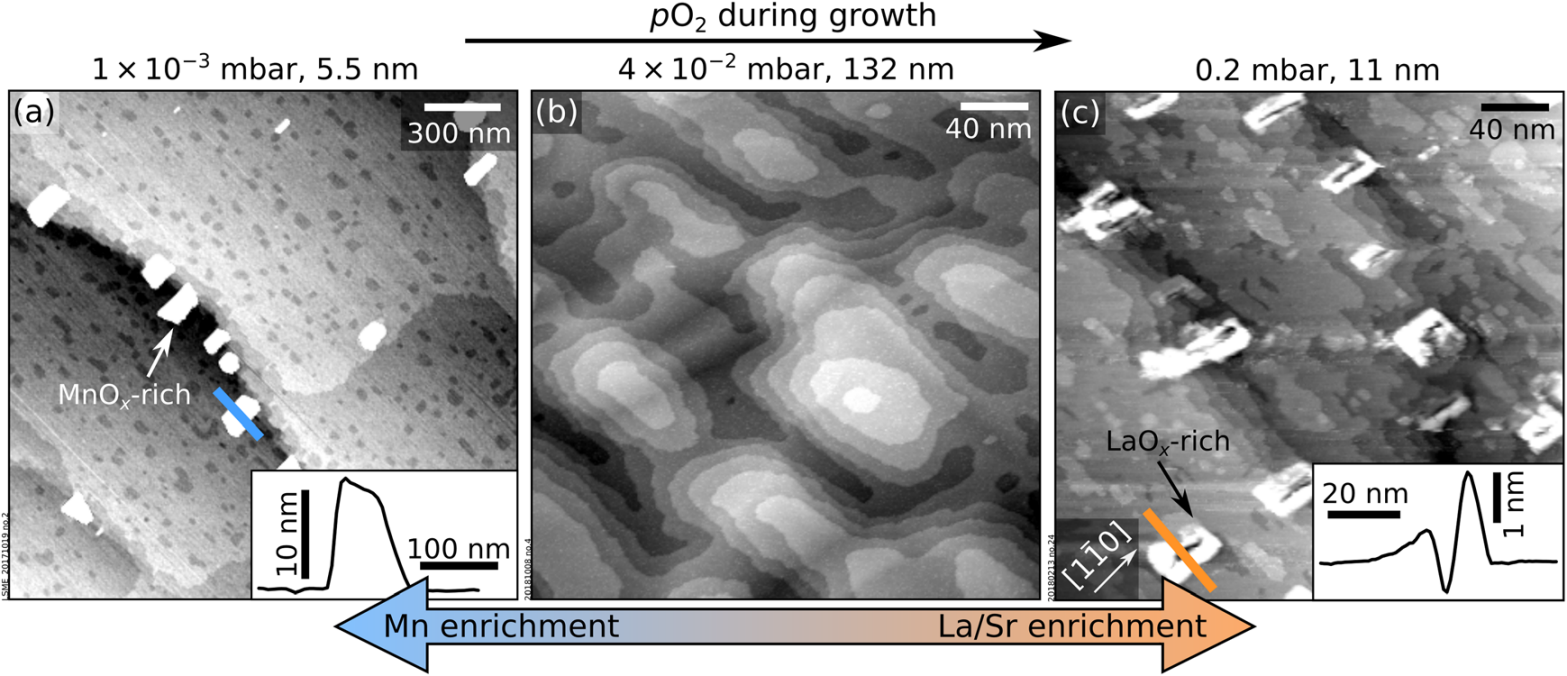
Accumulation of nonstoichiometry at the surface of LSMO(110) films. (a) 2.0 × 2.0 μm^2^ AFM image and (b and c) 300 × 300 nm^2^ STM images of LSMO(110) films thicker than those in Fig. 1 grown at different *p*_O_2__ and otherwise identical deposition parameters as to Fig. 1. Nonstoichiometric growth conditions (a and c) result in poorly conductive, few nanometers-high features on the surface that are identified as manganese- and lanthanum-oxide-rich clusters at low and high *p*_O_2__, respectively. Line scans over selected clusters are shown in the insets. (b) Ideal stoichiometry leads to films without precipitates even at a large thickness.

Growing thicker films at the same conditions as in Fig. 1a–c induces dramatic morphology changes, see Fig. 3. The lowest and highest pressures (5 × 10^−3^ mbar, Fig. 3a; 0.2 mbar, Fig. 3c) produce new features a few nanometres in height, located mainly at the step edges and poorly conductive, as judged by the behaviour of STM. At the intermediate pressure of 4 × 10^−2^ mbar, the surface preserves its flatness up to a thickness of 132 nm. Note that the imperfect films shown in Fig. 3 are significantly thinner: clusters appear at thicknesses of only 5.5 and 11 nm in Fig. 3a and c, respectively.

The bulk properties of films without clusters were characterized with RBS and XRD, see Fig. S1 and S2, ESI.† The films are stoichiometric and crystalline. RBS quantified the composition as (La_0.78±0.03_Sr_0.22±0.03_)_1.06±0.05_MnO_3_, close to the target's (La_0.79±0.02_Sr_0.21±0.02_)_0.96±0.08_MnO_3_. The analysis of XRD reciprocal-space maps^59–66^ reveals that the films are crystalline but only partially relaxed – by 34.7% along [11̄0] and 6% along [001]. This is expected for heteroepitaxial films under slight stress^46^ that relax by introducing misfit dislocations and forming mosaics. The residual deformation in the film in all three directions has an absolute value of less than 2 pm per unit cell. Best-fit lattice constants and angles are reported in Table S1 of the ESI.†

### Discussion

As mentioned in the Introduction, stoichiometric growth in PLD can be achieved following the optimization of many parameters,^12^ among others the oxygen background pressure and the laser fluence.^47^ As discussed below, both parameters are responsible for the growth behaviours summarized by Fig. 1 and 3.

Changes in the film composition as a function of *p*_O_2__ in multi-element oxides can be explained within the three-pressure-regimes framework^23,48,49^ (regardless of the plume composition right after the ablation, which can be affected by the laser fluence, see below). At low-enough *p*_O_2__, the ablated species are congruently transferred to the substrate. At intermediate pressures, lighter species are scattered more than heavier ones and the film becomes enriched with the heavier species as *p*_O_2__ increases.^50^ At very high *p*_O_2__, in the so-called shock-wave regime, all ablated species are slowed down equally; they are kept confined in the plume and transferred congruently to the substrate. These pressure regimes are identified not only by *p*_O_2__ but also by the target-to-substrate distance^16^*D* (for the experimental setup used here, *D* = 55 mm). Here, the low-pressure regime occurs at *p*_O_2__ ≤ 5 × 10^−3^ mbar: Below this value, there is no change in the XPS signals in Fig. 1, indicating congruent transfer. The intermediate pressure regime occurs at 3 × 10^−2^ ≤ *p*_O_2__ ≤ 0.2 mbar: the Mn content decreases with increasing pressure (Mn is the lightest cation in LSMO).

If the laser fluence is chosen such that all the species are ablated congruently at the target, the target has ideal stoichiometry, and the sticking probability of all ablated species on the surface is the same, it is possible to achieve near-ideal stoichiometries by depositing within either the low- or the high-pressure regimes. However, it is rare that all these conditions are fulfilled. Tiny deviations in the laser fluence or pulse duration can affect the ablation significantly, inducing the preferential ablation of one element over another in the multielement oxide. This is exemplified by the homoepitaxy of SrTiO_3_, where congruent ablation is achieved only within a very narrow window of laser fluence.^13,17,39,51^ Moreover, reproducing the laser fluence in different PLD setups is challenging:^13^ The spot size and the intensity distribution within the spot affect the deposition greatly;^12^ the most common way to adjust the UV pulse energy, by changing the discharge voltage of the UV laser, affects the pulse duration and beam divergences. Moreover, UV laser gases age over time, causing increasing pulse-to-pulse standard deviations and ill-defined fluences.

This work demonstrates how to achieve near-ideal stoichiometries even when the ablation is incongruent by exploiting the wider tunability window offered by the oxygen background pressure: the cation non-stoichiometry caused by preferential ablation can be mitigated by exploiting preferential scattering effects within the intermediate-pressure regime.

The “intermediate-pressure” film of Fig. 3b has a composition close to that of the target (see RBS analysis in Fig. S1c, ESI†). Then, according to the XPS data in Fig. 1d, films grown at lower and higher *p*_O_2__ (*i.e.*, congruent transfer regimes) must be Mn rich and Mn deficient, respectively. This means that Mn is preferentially ablated at the target with the chosen laser fluence: Mn-rich films are obtained at low *p*_O_2__ because the Mn-enriched plume is transferred congruently. The ideal stoichiometry is achieved at a specific value of intermediate *p*_O_2__ where the excess Mn is scattered more than the heavy La species. Finally, at even higher pressures, more than the excess Mn is scattered away and the films grow La-rich. The preferential ablation of Mn at the target is possibly caused by the laser fluence being too low. Previous studies on SrTiO_3_ homoepitaxy have shown that low fluences produce Sr-rich films,^52,53^ probably because of the higher vapor pressure of Sr compared to Ti; similarly, Mn could be preferentially ablated at low laser fluences because of its higher vapor pressure compared to La.

The different film compositions translate into different atomic-scale surface structures (Fig. 1a–c). The changes in the RHEED patterns of LSMO films grown at different *p*_O_2__ observed in the literature^22^ likely arise from the different surface reconstructions formed in each regime.

During SrTiO_3_(110) homoepitaxy, all non-stoichiometry segregates to the surface and changes its composition and atomic structure according to its composition phase diagrams.^13,39^ While it is not possible to prove that such full segregation occurs on LSMO(110) as well, several pieces of evidence support that segregation occurs at least partially, as seen by: (i) different surface structures forming on thin films with different compositions (Fig. 1), (ii) the evolution of the surface structures with increasing thickness (see Section 3), (iii) the XPS intensities mentioned in the previous paragraph, and (iv) the formation of non-conductive clusters when a critical thickness is overcome (Fig. 3). Since LSMO is an electrical conductor at room temperature, these clusters must consist of a different material. Hence, they are reasonably assigned to MnO_*x*_ and LaO_*x*_ excess introduced within the low- and high- pressure regimes, respectively. This is supported by the fact that similar clusters are observed after depositing large amounts (more than ≈2 ML) of Mn and La on well-defined LSMO(110) surfaces followed by short annealing times (≤20 min); and by the previously reported formation of MnO_*x*_ precipitates in epitaxial LSMO(001) films under Mn-rich conditions.^25^

The 3D clusters form when the surface cannot accommodate the excess cations by modifying its atomic structure. It was already reported that Mn (La) species stick less on Mn(La)-richer surfaces.^44^ A careful inspection of the LaO_*x*_ clusters in Fig. 3c reveals that they actually consist of pits surrounded by tall rims – similar to the features formed during the Ti-rich homoepitaxy of SrTiO_3_(110).^13^ There, the formation of pits was assigned to surface-dependent sticking and diffusion effects. The case of LSMO(110) appears similar. At La-rich conditions, the surface structure shifts towards La-rich reconstructions;^44^ at a critical composition, it becomes more favourable to nucleate and grow La-rich clusters than to incorporate more La in the surface structure.

Preferential sticking effects could act as a self-adjusting feedback mechanism for the film stoichiometry under slightly nonstoichiometric conditions: for slightly La-rich fluxes, the surface will gradually shift towards A-site richer reconstructions, onto which Mn sticks better, such that the surface shifts back towards Mn-richer structures. Now more La sticks, and so on. In principle, the forgiveness of this growth mechanism can allow growing atomically flat and stoichiometric films even under slightly nonstoichiometric conditions. However, as discussed above, this will work only up to a certain point: if the cation non-stoichiometry introduced by the incoming flux exceeds the capability of the surface to accommodate the non-stoichiometry *via* a change of the surface structure, oxide clusters form instead. One should also mention that, compared to SrTiO_3_(110), the surface reconstructions of LSMO(110) are separated by larger compositional differences.^39,44^ Hence, larger deposited non-stoichiometry can be accommodated at the surface of LSMO(110) films while yielding atomically flat films over a larger window of growth parameters.

One expects non-stoichiometry to accumulate at surfaces rather than in the bulk when forming bulk defects is comparatively more costly.^39,54^ Interestingly, the bulk phase diagrams of La_0.8_Sr_0.2_MnO_3_ (ref. 45) show that cation excesses are not easily incorporated in the bulk at UHV-compatible pressures. At atmospheric pressure, the system can accommodate significant excess of oxygen (and, possibly, cations). Nonetheless, our data show that excess Mn tends to float to the surface and change the surface atomic structure. Thus, non-stoichiometry segregation appears to be a powerful effect even in those perovskite oxides whose phase diagram would allow creating bulk defects.

## 3. Strategies to obtain ideal films

**Fig. 4:**
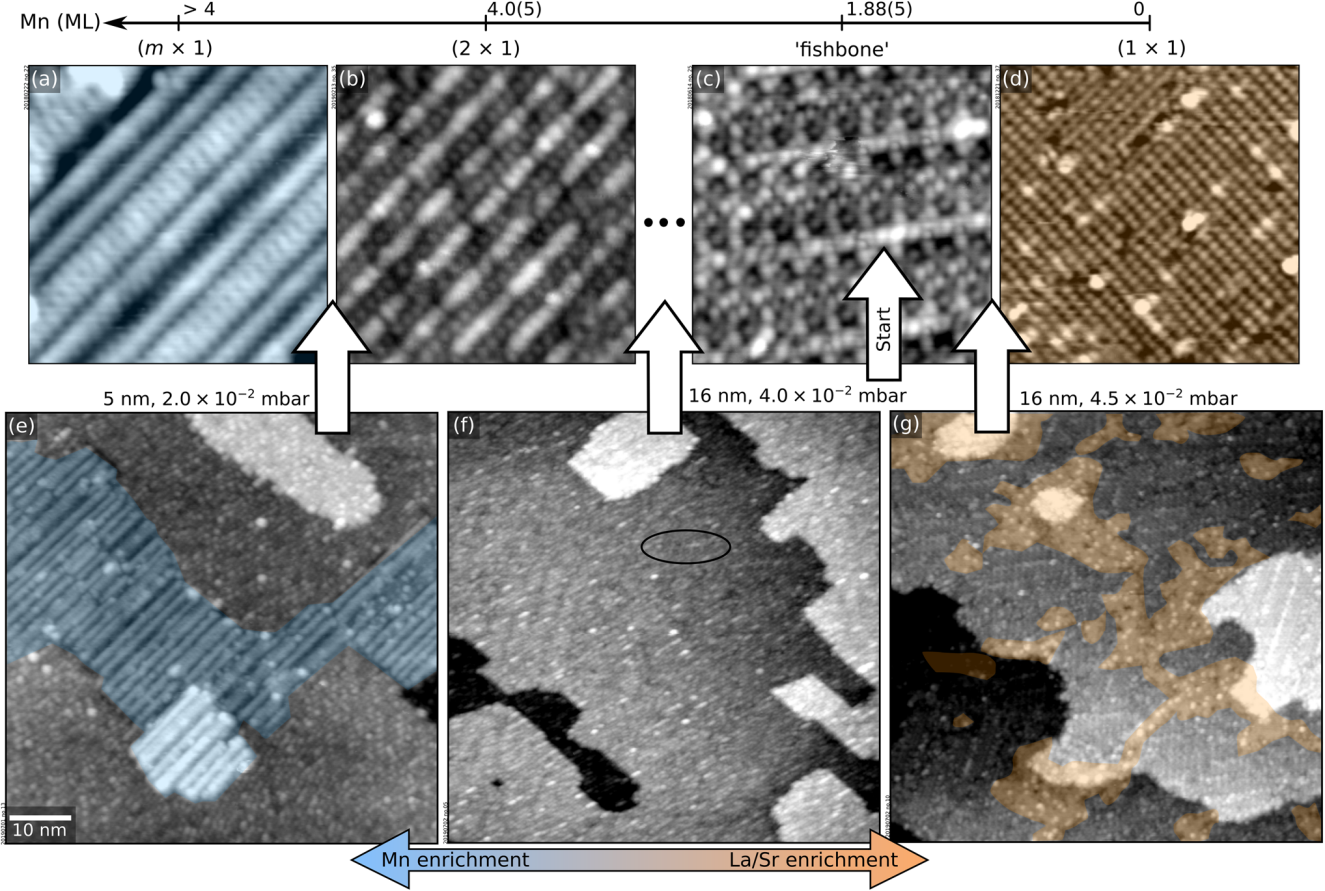
Pinpointing the exact *p*_O_2__ value for optimal LSMO(110) growth. (a–d) 12 × 12 nm^2^ STM images of selected surface structures of the surface phase diagram of LSMO(110),^44^ obtained by depositing controlled amounts of La or Mn from La_2_O_3_ and MnO targets in PLD plus annealing at 700 °C and 0.2 mbar O_2_ (see Methods section in the ESI;† 1 ML corresponds to the number of Mn sites in an (AMnO)_2_ plane of LSMO(110), *i.e.*, 4.64 × 10^14^ at. cm^−2^). (e–g) 70 × 70 nm^2^ STM images of LSMO films of various thicknesses grown at different *p*_O_2__, always starting from LSMO(110) films with the fishbone structure of panel (c). (e) 5 nm-thick film grown at 2.0 × 10^−2^ mbar O_2_, displaying patches of the Mn-rich (*m* × 1) structure of panel (a). (f) 16 nm-thick film grown at 4.0 × 10^−2^ mbar O_2_, with a surface reconstruction in between the fishbone and (2 × 1). (g) 16 nm-thick film grown at 4.5 × 10^−2^ mbar O_2_, with patches of the (1 × 1) and of the fishbone reconstructions.

This section illustrates a strategy to pinpoint the optimal growth conditions for LSMO(110) films (in this case, the value of *p*_O_2__ required to create a stoichiometric film in spite of the Mn-rich ablation). Several films were grown within the optimal range around 10^−2^ mbar (see Section 2). The corresponding surface structure changes were monitored with STM. Fig. 4 summarizes the results. For reference, the top row of Fig. 4 reports the surface phase diagram of LSMO(110) in the relevant *p*_O_2__ range.^44^ Before each deposition, the surface was prepared to exhibit the ‘fishbone’ reconstruction of Fig. 4c, *i.e.*, a 
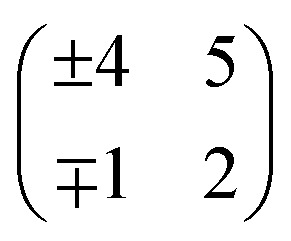
 superstructure. This reconstruction as well as the other surface structures and their short-hand notation are described in ref. 44.

First, 5 nm were grown at 2.0 × 10^−2^ mbar O_2_. The surface (Fig. 4e) is atomically flat but exhibits patches of the (*m* × 1) structure of Fig. 4a, indicating that this pressure introduces a significant Mn excess. Indeed, when continuing growth at this oxygen pressure, MnO_*x*_-rich clusters are formed at 10 nm thickness (not shown).

As learned from Fig. 1, higher values of *p*_O_2__ should introduce less Mn. By growing a film of 5 nm thickness at 4.0 × 10^−2^ mbar, the surface structure remains essentially the same as the starting point (not shown). Nonetheless, increasing the thickness to 16 nm (Fig. 4f) reveals a mix of the fishbone reconstruction (solid oval) and a reconstruction between the fishbone and the (2 × 1),^44^ indicating that the conditions are still Mn-enriching. Thus, the pressure should be increased further.

Growing 16 nm at *p*_O_2__ = 4.5 × 10^−2^ mbar on SrTiO_3_ pushes the surface toward the other end of the surface phase diagram (Fig. 4g): patches of the (1 × 1) reconstruction of Fig. 4d (A-site richer than the fishbone) coexist with fishbone-reconstructed areas. However, these conditions are still not ideal: larger thicknesses produce increasing areal coverages of (1 × 1) followed by the formation of AO_*x*_-rich clusters.

**Fig. 5:**
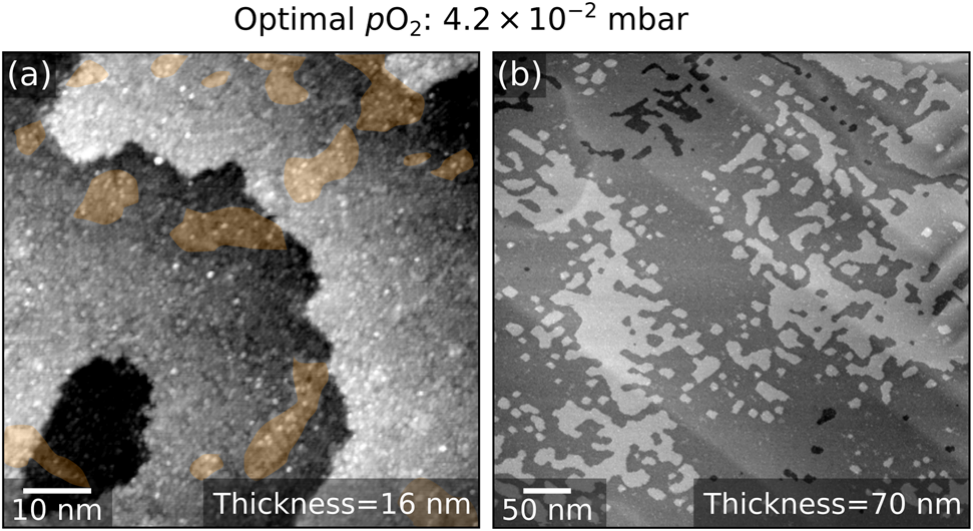
LSMO(110) film grown at optimized conditions. (a) 70 × 70 nm^2^ and (b) 500 × 500 nm^2^ STM images of 16 nm-thick and of 70 nm-thick LSMO(110) films, respectively, grown onto a fishbone-reconstructed LSMO(110) surface at 4.2 × 10^−2^ mbar O_2_. Both morphologies are atomically flat. At the atomic scale, (1 × 1) patches are visible [orange in panel (a)].

The ideal condition is reached at 4.2 × 10^−2^ mbar. A film of 16 nm thickness shows a small (1 × 1) coverage (Fig. 5a). A flat surface is maintained up to 70 nm thickness (Fig. 5b).

This section has shown how to grow flat films by detecting the non-stoichiometry from its influence on the surface reconstruction and adjusting *p*_O_2__ accordingly. Notably, it is also possible to recover films with precipitates by means of appropriate UHV treatments. Section S3 of ESI† shows that alternating annealing at oxidizing and reducing conditions favors surface diffusion and the flattening of the surface (as shown in ref. 55, this is a common behavior of oxide materials). This strategy is more effective than standard sputtering–annealing cycles.

## 4. Conclusions

This work addresses the correlation between non-stoichiometry (systematically tuned by varying the O_2_ background pressure), surface morphology, and surface atomic structures in PLD-grown LSMO(110) films by combining chemical analysis by XPS and atomically resolved STM. The often-overlooked surface atomic details have important implications for the growth. The surface can incorporate excess cations to a limited extent. In addition, the film composition may be influenced by preferential sticking. The composition-dependent surface atomic structures offer a precise metric to optimize the PLD parameters and achieve high-quality films. Since non-stoichiometries tend to segregate to the film surface and are prone to change its surface atomic structure, a stable atomic-scale surface structure at increasing film thicknesses indicates that the chosen PLD parameters yield a close-to-stoichiometric growth. On the other hand, a shift in the surface atomic structure indicates that cation excesses are introduced. To achieve close-to-stoichiometric films and avoid precipitation of undesired phases, the PLD parameters should be tuned such that the atomic-scale structure of the surface remains stable.

Monitoring the details of the surface atomic structures of the film also sheds light on previously disregarded mechanisms inducing morphological roughening. When the introduced non-stoichiometry exceeds a critical value, the surface cannot accommodate the excess cations by changing its atomic structure anymore. Instead, clusters of the excess material develop at the surface. In such cases, alternating annealing treatments at oxidizing and reducing conditions is an effective means to remediate the surface morphology.

Many phenomena observed during the growth of LSMO(110), including non-stoichiometry segregation that alters the surface structure, surface-dependent incorporation of deposited cations, and phase separation, seem to be a general trait of perovskite oxides and possibly many other multi-element compounds.^13,14,56,57^ A growing number of systems is reported to exhibit composition-related surface reconstructions with an apparent tendency to accommodate cation excesses. The authors contend that the findings reported here reflect general behaviours of complex oxide films, independent of the growth technique. The insights and methods presented can guide the growth optimization of perovskite-oxide films.

## Author contributions

Conceptualization: GF and MR. Investigation: GF, MR and RH. Validation: MR, MS and UD. Supervision: MR, MS and UD. Funding acquisition: UD. Writing, original draft: GF. Writing, review and editing: all authors.

## Conflicts of interest

There are no conflicts to declare.

## Supplementary Material

NA-005-D3NA00847A-s001

## References

[cit1] Zubko P., Gariglio S., Gabay M., Ghosez P., Triscone J. M. (2011). Interface physics in complex oxide heterostructures. Annu. Rev. Condens. Matter Phys..

[cit2] Bhalla A. S., Guo R., Roy R. (2000). The perovskite structure – A review of its role in ceramic science and technology. Mater. Res. Innovations.

[cit3] Peña M. A., Fierro J. L. G. (2001). Chemical structures and performance of perovskite oxides. Chem. Rev..

[cit4] Kumah D. P., Ngai J. H., Kornblum L. (2020). Epitaxial oxides on semiconductors: from fundamentals to new devices. Adv. Funct. Mater..

[cit5] Schlom D. G., Chen L. Q., Pan X., Schmehl A., Zurbuchen M. A. (2008). A thin film approach to engineering functionality into oxides. J. Am. Ceram. Soc..

[cit6] Junquera J., Ghosez P. (2003). Critical thickness for ferroelectricity in perovskite ultrathin films. Nature.

[cit7] Liao Z., Zhang J. (2019). Metal-to-insulator transition in ultrathin manganite heterostructures. Appl. Sci..

[cit8] Hemberger J., Krimmel A., Kurz T., Krug von Nidda H. A., Ivanov V. Y., Mukhin A. A., Balbashov A. M., Loidl A. (2002). Structural, magnetic, and electrical properties of single-crystalline La_1−*x*_Sr_*x*_MnO_3_ (0.4 < *x* < 0.85). Phys. Rev. B: Condens. Matter Mater. Phys..

[cit9] Sun C., Hui R., Roller J. (2010). Cathode materials for solid oxide fuel cells: A review. J. Solid State Electrochem..

[cit10] Hwang J., Rao R. R., Giordano L., Katayama Y., Yu Y., Shao-Horn Y. (2017). Perovskites in catalysis and electrocatalysis. Science.

[cit11] Ramesh R., Schlom D. G. (2019). Creating emergent phenomena in oxide superlattices. Nat. Rev. Mater..

[cit12] Ojeda-G-P A., Döbeli M., Lippert T. (2018). Influence of plume properties on thin film composition in pulsed laser deposition. Adv. Mater. Interfaces.

[cit13] Riva M., Franceschi G., Schmid M., Diebold U. (2019). Epitaxial growth of complex oxide films: Role of surface reconstructions. Phys. Rev. Res..

[cit14] Schöffmann P., Pütter S., Schubert J., Zander W., Barthel J., Zakalek P., Waschk M., Heller R., Brückel T. (2020). Tuning the Co/Sr stoichiometry of SrCoO_2.5_ thin films by RHEED assisted MBE growth. Mater. Res. Express.

[cit15] Schraknepper H., Bäumer C., Gunkel F., Dittmann R., De Souza R. A. (2016). Pulsed laser deposition of SrRuO_3_ thin-films: The role of the pulse repetition rate. APL Mater..

[cit16] Koubaa M., Haghiri-Gosnet A. M., Desfeux R., Lecoeur P., Prellier W., Mercey B. (2003). Crystallinity, surface morphology, and magnetic properties of La_0.7_Sr_0.3_MnO_3_ thin films: an approach based on the laser ablation plume range models. J. Appl. Phys..

[cit17] Ohnishi T., Koinuma H., Lippmaa M. (2006). Pulsed laser deposition of oxide thin films. Appl. Surf. Sci..

[cit18] Bachelet R., Pesquera D., Herranz G., Sánchez F., Fontcuberta J. (2010). Persistent two-dimensional growth of (110) manganite films. Appl. Phys. Lett..

[cit19] Guo H., Sun D., Wang W., Gai Z., Kravchenko I., Shao J., Jiang L., Ward T. Z., Snijders P. C., Yin L., Shen J., Xu X. (2013). Growth diagram of La_0.7_Sr_0.3_MnO_3_ thin films using pulsed laser deposition. J. Appl. Phys..

[cit20] Annese E., Mori T. J. A., Schio P., Salles B. R., Cezar J. C. (2018). Influence of the growth parameters on the electronic and magnetic properties of La_0.67_Sr_0.33_MnO_3_ epitaxial thin films. Appl. Surf. Sci..

[cit21] Ma J. X., Liu X. F., Lin T., Gao G. Y., Zhang J. P., Wu W. B., Li X. G., Shi J. (2009). Interface ferromagnetism in (110)-oriented La_0.7_Sr_0.3_MnO_3_/SrTiO_3_ ultrathin superlattices. Phys. Rev. B: Condens. Matter Mater. Phys..

[cit22] Huijben M., Martin L. W., Chu Y.-H., Holcomb M. B., Yu P., Rijnders G., Blank D. H. A., Ramesh R. (2008). Critical thickness and orbital ordering in ultrathin La_0.7_Sr_0.3_MnO_3_ films. Phys. Rev. B: Condens. Matter Mater. Phys..

[cit23] Chen J., Döbeli M., Stender D., Conder K., Wokaun A., Schneider C. W., Lippert T. (2014). Plasma interactions determine the composition in pulsed laser deposited thin films. Appl. Phys. Lett..

[cit24] Chaluvadi S. K., Polewczyk V., Petrov A. Y., Vinai G., Braglia L., Diez J. M., Pierron V., Perna P., Mechin L., Torelli P., Orgiani P. (2022). Electronic properties of fully strained La_1-*x*_Sr_*x*_MnO_3_ thin films grown by molecular beam epitaxy (0.15 ≤ *x* ≤ 0.45). ACS Omega.

[cit25] Steffen A., Glavic A., Gutberlet T., Ambaye H., Schubert J., Geprägs S., Barthel J., Mattauch S., Zander W., Kruth M., Schöffmann P., Pütter S., Brückel T. (2021). Unexpected precipitates in conjunction with layer-by-layer growth in Mn-enriched La_2/3_Sr_1/3_MnO_3_ thin films. Thin Solid Films.

[cit26] Ishii Y., Sato H., Sawa A., Yamada T., Akoh H., Endo K., Kawasaki M., Tokura Y. (2004). Precipitate-free films of La_1-*x*_Sr_*x*_MnO_3_ grown on the substrates with artificial step edges. Appl. Phys. Lett..

[cit27] Schmid M., Lenauer C., Buchsbaum A., Wimmer F., Rauchbauer G., Scheiber P., Betz G., Varga P. (2009). High island densities in pulsed laser deposition: Causes and implications. Phys. Rev. Lett..

[cit28] Jeffries J. H., Zuo J. K., Craig M. M. (1996). Instability of kinetic roughening in sputter-deposition growth of Pt on glass. Phys. Rev. Lett..

[cit29] Spencer B. J., Davis S. H., Voorhees P. W. (1993). Morphological instability in epitaxially strained dislocation-free solid films: Nonlinear evolution. Phys. Rev. B: Condens. Matter Mater. Phys..

[cit30] Palmer B. J., Gordon R. G. (1989). Kinetic model of morphological instabilities in chemical vapor deposition. Thin Solid Films.

[cit31] Jamnig A., Sangiovanni D. G., Abadias G., Sarakinos K. (2019). Optimization of La_0.7_Ba_0.3_MnO_3-*δ*_ complex oxide laser ablation conditions by plume imaging and optical emission spectroscopy. Sci. Rep..

[cit32] Zepeda-Ruiz L. A., Gilmer G. H., Walton C. C., Hamza A. V., Chason E. (2010). Surface morphology evolution during sputter deposition of thin films – lattice Monte Carlo simulations. J. Cryst. Growth.

[cit33] Kienzle D., Koirala P., Marks L. D. (2015). Lanthanum aluminate (110) 3×1 surface reconstruction. Surf. Sci..

[cit34] Levchenko S. V., Rappe A. M. (2008). Influence of ferroelectric polarization on the equilibrium stoichiometry of lithium niobate (0001) surfaces. Phys. Rev. Lett..

[cit35] Gerhold S., Wang Z., Schmid M., Diebold U. (2014). Stoichiometry-driven switching between surface reconstructions on SrTiO_3_(001). Surf. Sci..

[cit36] Kolpak A. M., Li D., Shao R., Rappe A. M., Bonnell D. A. (2008). Evolution of the structure and thermodynamic stability of the BaTiO_3_(001) surface. Phys. Rev. Lett..

[cit37] Saidi W. A., Martirez J. M. P., Rappe A. M. (2014). Strong reciprocal interaction between polarization and surface stoichiometry in oxide ferroelectrics. Nano Lett..

[cit38] Russell B. C., Castell M. R. (2008). Surface of sputtered and annealed polar SrTiO_3_(111): TiO_*x*_-rich (*n* × *n*) reconstructions. J. Phys. Chem. C.

[cit39] Riva M., Franceschi G., Lu Q., Schmid M., Yildiz B., Diebold U. (2019). Pushing the detection of cation nonstoichiometry to the limit. Phys. Rev. Mater..

[cit40] Franceschi G., Schmid M., Diebold U., Riva M. (2021). Two-dimensional surface phase diagram of a multicomponent perovskite oxide: La_0.8_Sr_0.2_MnO_3_(110). Phys. Rev. Mater..

[cit41] Kajdos A. P., Stemmer S. (2014). Surface reconstructions in molecular beam epitaxy of SrTiO_3_. Appl. Phys. Lett..

[cit42] Sánchez F., Ocal C., Fontcuberta J. (2014). Tailored surfaces of perovskite oxide substrates for conducted growth of thin films. Chem. Soc. Rev..

[cit43] Chang Y. J., Phark S.-H. (2017). Atomic-scale visualization of initial growth of perovskites on SrTiO_3_(001) using scanning tunneling microscope. Curr. Appl. Phys..

[cit44] Franceschi G., Schmid M., Diebold U., Riva M. (2020). Atomically resolved surface phases of La_0.8_Sr_0.2_MnO_3_(110) thin films. J. Mater. Chem. A.

[cit45] Grundy A. N., Hallstedt B., Gauckler L. J. (2004). Assessment of the La–Sr–Mn–O system. Calphad.

[cit46] Franceschi G., Wagner M., Hofinger J., Krajňák T., Schmid M., Diebold U., Riva M. (2019). Growth of In_2_O_3_(111) thin films with optimized surfaces. Phys. Rev. Mater..

[cit47] Wicklein S., Sambri A., Amoruso S., Wang X., Bruzzese R., Koehl A., Dittmann R. (2012). Pulsed laser ablation of complex oxides:
The role of congruent ablation and preferential scattering for the film stoichiometry. Appl. Phys. Lett..

[cit48] Amoruso S., Aruta C., Bruzzese R., Maccariello D., Maritato L., Granozio F. M., Orgiani P., di Uccio U., Wang X. (2010). Optimization of La_0.7_Ba_0.3_MnO_3−*δ*_ complex oxide laser ablation conditions by plume imaging and optical emission spectroscopy. J. Appl. Phys..

[cit49] Orgiani P., Ciancio R., Galdi A., Amoruso S., Maritato L. (2010). Physical properties of La_0.7_Ba_0.3_MnO_3−*δ*_ complex oxide thin films grown by pulsed laser deposition technique. Appl. Phys. Lett..

[cit50] Tyunina M., Levoska J., Leppävuori S. (1998). Experimental studies and modeling of Pb-Zr-Ti-O film growth in pulsed laser deposition. J. Appl. Phys..

[cit51] Liu G. Z., Lei Q. Y., Xi X. X. (2012). Stoichiometry of SrTiO_3_ films grown by pulsed laser deposition. Appl. Phys. Lett..

[cit52] Ohnishi T., Shibuya K., Yamamoto T., Lippmaa M. (2008). Defects and transport in complex oxide thin films. J. Appl. Phys..

[cit53] Dam B., Rector J. H., Johansson J., Huijbregtse J., De Groot D. G. (1998). Mechanism of incongruent ablation of SrTiO_3_. J. Appl. Phys..

[cit54] Enterkin J. A., Subramanian A. K., Russell B. C., Castell M. R., Poeppelmeier K. R., Marks L. D. (2010). A homologous series of structures on the surface of SrTiO_3_(110). Nat. Mater..

[cit55] Franceschi G., Schmid M., Diebold U., Riva M. (2022). Reconstruction changes drive surface diffusion and determine the flatness of oxide surfaces. J. Vac. Sci. Technol., A.

[cit56] Brahlek M., Sen Gupta A., Lapano J., Roth J., Zhang H. T., Zhang L., Haislmaier R., Engel-Herbert R. (2018). Frontiers in the growth of complex oxide thin films: past, present, and future of hybrid MBE. Adv. Funct. Mater..

[cit57] Rachut K., Bayer T. J. M., Wolff J. O., Kmet B., Benčan A., Klein A. (2019). Off-stoichiometry of magnetron sputtered Ba_1−*x*_Sr_x_TiO_3_ thin films. Phys. Status Solidi B.

[cit58] Gerhold S., Riva M., Yildiz B., Schmid M., Diebold U. (2016). Adjusting island density and morphology of the SrTiO_3_(110)-(4×1) surface: Pulsed laser deposition combined with scanning tunneling microscopy. Surf. Sci..

[cit59] Kriegner D., Wintersberger E., Stangl J. (2013). *xrayutilities*: A versatile tool for reciprocal space conversion of scattering data recorded with linear and area detectors. J. Appl. Crystallogr..

[cit60] Mebane D. S., Liu Y., Liu M. (2008). Refinement of the bulk defect model for La*_x_*Sr_1−*x*_MnO_3±*δ*_. Solid State Ionics.

[cit61] Dong C., Wu F., Chen H. (1999). Correction of zero shift in powder diffraction patterns using the reflection-pair method. J. Appl. Crystallogr..

[cit62] Megaw H. D., Darlington C. N. W. (1975). Geometrical and structural relations in the rhombohedral perovskites. Acta Crystallogr., Sect. A: Cryst. Phys., Diffr., Theor. Gen. Crystallogr..

[cit63] Darling T., Migliori A., Moshopoulou E., Trugman S. A., Neumeier J., Sarrao J., Bishop A., Thompson J. (1998). Measurement of the elastic tensor of a single crystal of La_0.83_Sr_0.17_MnO_3_ and its response to magnetic fields. Phys. Rev. B: Condens. Matter Mater. Phys..

[cit64] Marcus P. M., Jona F. (1995). Strains in epitaxial films: The general case. Phys. Rev. B: Condens. Matter Mater. Phys..

[cit65] Virtanen P., Gommers R., Oliphant T. E., Haberland M., Reddy T., Cournapeau D., Burovski E., Peterson P., Weckesser W., Bright J., van der Walt S. J., Brett M., Wilson J., Millman K. J., Mayorov N., Nelson A. R. J., Jones E., Kern R., Larson E., Carey C. J., Polat İ., Feng Y., Moore E. W., VanderPlas J., Laxalde D., Perktold J., Cimrman R., Henriksen I., Quintero E. A., Harris C. R., Archibald A. M., Ribeiro A. H., Pedregosa F., van Mulbregt P., SciPy 1.0 Contributors (2020). SciPy 1.0: fundamental algorithms for scientific computing in Python. Nat. Methods.

[cit66] Gražulis S., Chateigner D., Downs R. T., Yokochi A. F. T., Quirós M., Lutterotti L., Manakova E., Butkus J., Moeck P., Le Bail A. (2009). Crystallography Open Database – An open-access collection of crystal structures. J. Appl. Crystallogr..

