# Supplementary material for: Evolution of the surface atomic structure of multielement oxide films: curse or blessing?
Source: Nanoscale Adv. 2023 Nov 2;5(24):7009–17. doi: 10.1039/d3na00847a (PMC10696924; doi:10.1039/d3na00847a)
Supplement: NA-005-D3NA00847A-s001 [file NA-005-D3NA00847A-s001.pdf]

Electronic Supplementary Material (ESI) for Nanoscale Advances.
This journal is © The Royal Society of Chemistry 2023

Electronic Supplementary Information (ESI)

Evolution of the surface atomic structure of multielement oxide films: curse or blessing?

Giada Franceschi, Renè Heller, Michael Schmid, Ulrike Diebold, and Michele Riva

S1. Materials and methods

The LSMO/$SrTiO_3$(110) films were grown in a ultra-high vacuum pulsed-laser deposition (UHV PLD) setup (base pressure $< 4 \times 10^{-10}$ mbar after bake-out) fitted for high-pressure and high-temperature growth experiments. This PLD setup is attached via an intermediate UHV chamber to a surface characterization facility (base pressure below 4×10^{-11} mbar) comprising scanning tunneling microscopy (STM), low-energy electron diffraction (LEED), x-ray photoelectron spectroscopy (XPS), and low-energy He^+-ion scattering (LEIS). The UHV system and all technical details about the growth of LSMO/$SrTiO_3$(110) films are discussed elsewhere.[1,2]

The $SrTiO_3$(110) substrates (single crystals from CrysTec GmbH, 0.5 wt.% Nb-doped, $5 \times 5 \times 0.5$ mm^3, one-side polished, miscut < 0.3°) were prepared and characterized in UHV to exhibit a mixture of (4 × 1) and (5 × 1) surface reconstructions[3] (checked in STM; purity checked in XPS before growth).

The LSMO films were grown at 1 Hz laser repetition rate, laser fluence of 1.9 J/cm^2, 700 °C substrate temperature, and O_2 pressures between 7×10^{-6} mbar and 0.2 mbar. The surface composition and atomic structure of some films used as a substrate for further deposition were tuned by PLD of controlled amounts of La and Mn from La_2O_3 and MnO targets (2 Hz, 1.5 J/cm^2, 0.2 mbar O_2, RT), followed by annealing for at least 30 min at 700 °C, 0.2 mbar O_2.[1] The thickness was estimated by monitoring the evolution of the specular-spot intensity of RHEED (in the layer-by-layer growth mode one oscillation corresponds to one cation layer of ≈ 0.28 nm; see Fig. 2 in the main text). After growth the films were moved in UHV to the analysis chamber for the STM and XPS measurements.

STM images were acquired at room temperature, in constant-current mode, and measuring empty states (positive sample bias V_{sample}). Electrochemically etched W tips were prepared by Ar^+ sputtering. Sometimes it helped to indent the tip into the film or to apply current/voltage pulses. After such tip preparation steps, STM images were acquired on a different spot of the sample.

XPS data were acquired in normal emission with a non-monochromatic Al Kα source and a SPECS Phoibos 100 analyser. The areas of Mn 2*p*, La 4*d*, Sr 3*d* XPS core-level peaks were evaluated with CasaXPS after subtracting a Shirley-type background.

Sputtering was performed with Ar^+ ions with 1 keV energy, 45° incidence, and sputter current density of ≈ 6 µA/cm^2, corresponding to ≈ 0.4 ions/nm^2 s (without correction for secondary electrons).

XRD data were collected at the TU Wien X-ray Centre [PANalytical Empyrean; Cu Kα_1 radiation obtained with a 2-pass Ge(220) hybrid monochromator and a 1/32° anti-divergence slit; a GaliPIX3D area detector with 0.02 rad Soller slits was used to measure reciprocal-space maps]. They were analysed with the xrayutilities Python package.[4]

For RBS, a 1.7 MeV $^4He^+$ ion beam was directed under normal incidence onto the sample, while the detector (resolution of 17 keV) was mounted under a backscattering angle of 170°. The total charge deposited was 10–20 µC (values determined by fitting the bulk signal for each spectrum). Areal densities and layer compositions were determined with the simulation code SIMNRA.

S2. RBS and XRD characterization of atomically flat films

Figures S1 and S2 summarize the bulk characterization analysis (RBS and XRD) performed on cluster-free LSMO(110) films. RBS (Fig. S1) quantified the film stoichiometry as $(La_{0.78 \pm 0.03}Sr_{0.22 \pm 0.03})_{1.06 \pm 0.05}MnO_3$, close to the composition of the target $(La_{0.79 \pm 0.02}Sr_{0.21 \pm 0.02})_{0.96 \pm 0.08}MnO_3$ (errors represent one standard deviation). The target composition was measured by

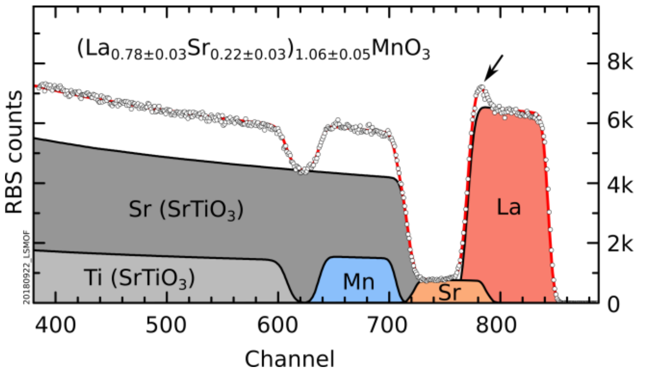

Fig. S1. RBS characterization of a $La_{0.8}Sr_{0.2}MnO_3$(110) film with 132 nm thickness grown with appropriate conditions (= 4.2×10^{-2} mbar, and otherwise identical parameters as reported in the main text) to render an ideal surface morphology. Experimental data (dots) are fit well (red line) by the contribution of the $SrTiO_3$ substrate (grey) and LSMO (coloured). Fitting of the RBS spectrum yields a composition of $(La_{0.78 \pm 0.03}Sr_{0.22 \pm 0.03})_{1.06 \pm 0.05}MnO_3$, close to the composition of the LSMO target, $(La_{0.79 \pm 0.02}Sr_{0.21 \pm 0.02})_{0.96 \pm 0.08}MnO_3$.[1] The bump indicated by the arrow does not correspond to accumulation of La at the interface; instead, it originates from the overlap of the La and Sr signals of LSMO.

inductively coupled-plasma mass spectrometry — ICP-MS. Note that the bump indicated by the arrow in Fig. S1 results from the overlap of the La and Sr signals of LSMO. The experimental data (dots) were fitted (red solid line) by the sum of the individual components of the $SrTiO_3$ substrate and LSMO film.

It is worth to point out that the RBS analysis is not sufficient to claim a perfect composition of the films, especially with the given error bars. This analysis supports our findings that the stoichiometry of the film fits in the expected range, and that no significant cation excesses are introduced into the bulk while the surface atomic structure remains stable at increasing thicknesses.

Other techniques (XPS, transmission electron microscopy – TEM, ICP-MS) were attempted but did not deliver reliable stoichiometry estimates. XPS suffered from forward-focusing effects and the lack of reliable reference samples for the single elements. In TEM, the preferential removal of Mn by Ar^+ milling while making the lamella produced different compositions as a function of the lamella's thickness. ICP-MS was problematic because of preferential cation dissolution and the presence of Sr in both film and substrate. Note that the oxygen stoichiometry of the films cannot be determined by RBS. Nonetheless, Kröger-Vink defect diagrams of 20%-doped LSMO[5] show that, at all experimental conditions presented in this work, the films should be oxygen-stoichiometric. At 10^{-6} mbar (the most reducing O_2 pressure used in our work) and 800 °C (i.e., 100 °C higher than our growth temperature; hence, more reducing), the concentration of oxygen vacancies per oxygen sites is at most 10^{-5}.

Figure S2 shows XRD reciprocal-space maps measured for both symmetric (left column) and asymmetric reflexes. Asymmetric reflexes were measured with either the $[1\bar{1}0]$ or the $[00\bar{1}]$ directions of $SrTiO_3$ in the scattering plane, in both grazing incidence and grazing emission geometries. All panels show reflexes from the $SrTiO_3$ substrate (black dots) and the LSMO film (orange).

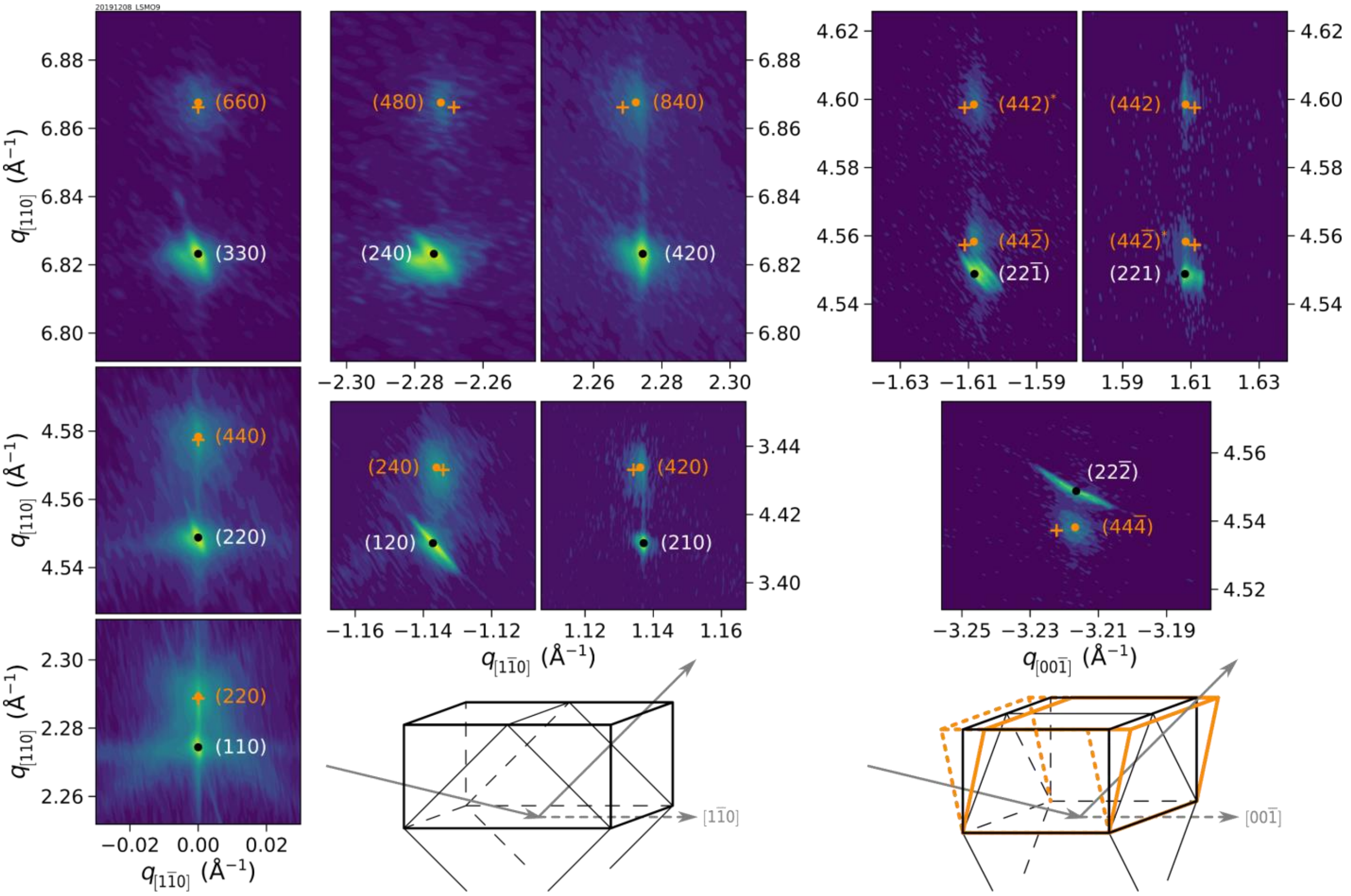

Fig. S2. XRD characterization of a 70 nm-thick $La_{0.8}Sr_{0.2}MnO_3(110)$ film grown with appropriate conditions to obtain an ideal surface morphology (= 4.2 × 10^{-2} mbar, and otherwise identical parameters as reported in the main text). Reciprocal-space maps acquired around symmetric (left column) and asymmetric (remaining panels) reflexes. Intensities are plotted with a logarithmic colour scale normalized to the intensity of the $SrTiO_3$ reflex in each panel. $q_{[110]}$ is the component of the momentum transfer perpendicular to the surface; $q_{[1\bar{1}0]}$ and $q_{[00\bar{1}]}$ are components in the surface plane. Negative in-plane components represent grazing-incidence configurations. $q = 2\pi/d$ when d is the distance between diffracting planes. Black dots and white labels mark the position of reflexes of the $SrTiO_3$ substrate. Orange dots mark the position of reflexes of the best-fit structure for the LSMO film (see text). Orange crosses identify the positions for fully relaxed LSMO. Reflexes from two mirror-symmetric LSMO domains are visible in the rightmost panels (marked with an asterisk; also see the two domains schematically drawn in orange in the lower-right sketch). The measurement geometries are sketched at the bottom.

This journal is © The Royal Society of Chemistry 2023

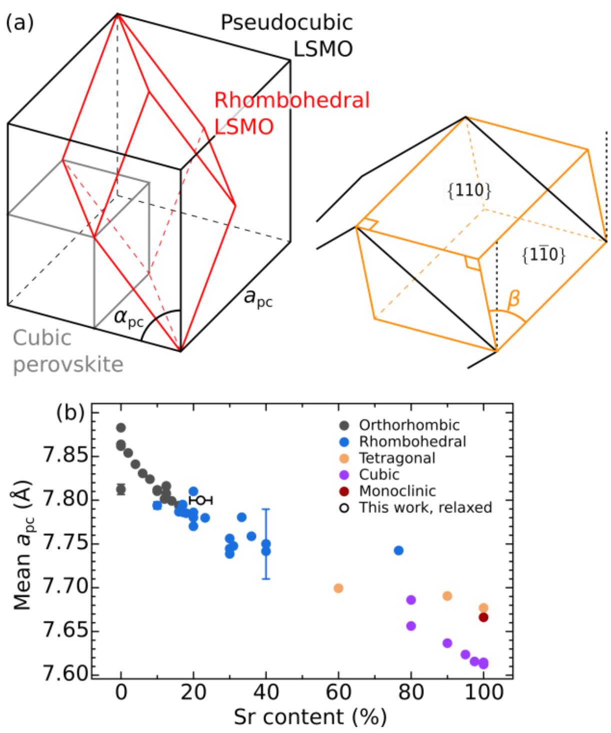

Fig. S3. (a) Sketches of the primitive (rhombohedral, red) and pseudocubic (black) unit cells of LSMO. In grey is the unit cell of a cubic perovskites such as $SrTiO_3$. In orange is the monoclinic unit cell bound by the $\{110\}$, and $\{1\bar{1}0\}$ planes of the pseudocubic unit cell. Notice that in this monoclinic cell, the $\{1\bar{1}0\}$ plane is non-rectangular. (b) Lattice constants of pseudocubic unit cells for LSMO as a function of the Sr content derived from the Crystallography Open Database.[12] For tetragonal, orthorhombic, and monoclinic primitive cells, the values plotted are a mean of the lattice parameters in the three directions.

Multiple $SrTiO_3$ maxima are visible [see especially the $(22\bar{1})$ and (221) reflexes], as already observed on other commercial single crystals.[3] LSMO reflexes are broader than those of $SrTiO_3$, as expected for heteroepitaxial films under slight stress[6] that relax by introducing misfit dislocations and forming mosaics. Signs of this relaxation are also visible in the large-area STM images as long-range sub-unit-cell-height contrast modulations (see, e.g., Figs. 3b and 5b of the main text).

The raw XRD data were corrected for small offsets of the machine readings using the $SrTiO_3$ reflexes: the 2Θ offset was calculated by exploiting the known ratios of interplanar spacings for given (hkl) pairs [e.g., the separation of (110) and (220) is one third of the separation of (110) and (330)];[7] the ω offset was derived from the known ratios of in- and out-of-plane components of reciprocal-space vectors $\mathbf{q}_{hkl}$. Both adjustments are independent of the lattice constants. To appropriately fit the offset-corrected $SrTiO_3$ reflexes, it was necessary to use a lattice constant of 3.90685(41) Å, deviating about 0.05% from the nominal constant of 3.905 Å.

To explain the LSMO reflexes, one must take the correct unit cell of LSMO. At the 80:20 La:Sr composition, LSMO has a rhombohedral primitive unit cell with $R\bar{3}c$ space group (red in Fig. S3a). When grown on a cubic perovskite like $SrTiO_3$, it is more convenient to transform this rhombohedral cell to a non-primitive pseudocubic (pc) one with side $a_{pc} \approx 8$ Å and $\alpha_{pc} = 90° + \delta$ (black in Fig. S3a; approximately corresponding to a $2 \times 2 \times 2$ supercell of the cubic perovskite unit cell, grey in Fig. S3a).[1,8] The (hkl) indices for LSMO reported in Fig. S2 correspond to this pseudocubic unit cell. In the pseudocubic cell, the $\{110\}$ and $\{1\bar{1}0\}$ facets are inequivalent. The unit cell bound by the pseudocubic $\{110\}$ and $\{1\bar{1}0\}$ facets is monoclinic (orange in Fig. S3a) with $\cos\beta = \cos\alpha_{pc} / \cos(\alpha_{pc}/2)$. The $\{110\}$ plane has a rectangular 2D unit cell, while $\{1\bar{1}0\}$ is non-rectangular [angles β and $(180° - \beta)$]. In principle, both orientations could be present after film growth. If the film grows $\{110\}$-oriented, the rectangular $\{110\}$ plane lies parallel to the surface of the $SrTiO_3(110)$ substrate, and the out-of-plane lattice vector is oriented at an angle β. Conversely, $\{1\bar{1}0\}$-oriented films have their non-rectangular face on $SrTiO_3(110)$, and the rectangular one perpendicular to the surface. The XRD data in Fig. S2 show that the film is purely $\{110\}$-oriented. This is evidenced by the panels in the right column of Fig. S2, showing a set of two reflexes from LSMO at the positions expected for $\{110\}$-oriented films (one marked with an asterisk). The presence of two reflexes, (442) and $(44\bar{2})$, is expected because the out-of-plane tilt by β breaks the mirror symmetry of $SrTiO_3$ and results in two symmetry-inequivalent domains. Instead, there would be only one set of spots for $\{1\bar{1}0\}$-oriented films roughly midway those in the right column of Fig. S2. The exclusive presence of $\{110\}$ — as opposed to the coexistence of $\{110\}$ and $\{1\bar{1}0\}$ — is reasonable since $\{1\bar{1}0\}$ should be energetically unfavourable: it requires additional shear stress to distort its in-plane non-rectangular cell and match the $SrTiO_3(110)$ mesh.

To fit the LSMO reflexes, the following free independent parameters were considered: the lattice parameter a_{pc} and angle α_{pc} of the unstrained (fully relaxed) pseudocubic cell, and the amount of relaxation of the misfit strain along the $[1\bar{1}0]$ and $[001]$ in-plane directions of $SrTiO_3$. For the fit, the relaxed pseudocubic LSMO cell was placed onto $SrTiO_3(110)$; in-plane misfit strain was applied including relaxation; out-of-plane strain was derived using the known elastic constants of LSMO[9] following the approach of Ref. 10. The distance in reciprocal space between experimental and calculated **q**-vectors was minimized using the differential evolution algorithm implemented in the scipy Python library.[11]

Table S1. Lattice constants (expressed in ångstroms) and monoclinic angles arising from the analysis of the XRD data.

	In-plane		Out-of-plane	
	$[001]$	$[1\bar{1}0]$	$[110]$	β(°)
Relaxed	7.7998	5.5394	5.4910	90.714
Strained	7.8129	5.5301	5.4899	90.716
$SrTiO_3$	7.8137	5.5251	5.5251	90.000

The fitting procedure yielded the results summarized in Table S1. The unstrained LSMO (orange crosses in Fig. S2) has a lattice constant of $a_{pc} = 7.7998$ Å and an angle of $\alpha_{pc} = 90.503°$. These values are in perfect agreement with the trend of lattice constants of LSMO as a function of the La:Sr ratio derived from the Crystallography Open Database (Fig. S3b).[12] The film is only partially relaxed (by 6% along $[001]$ and 34.7%

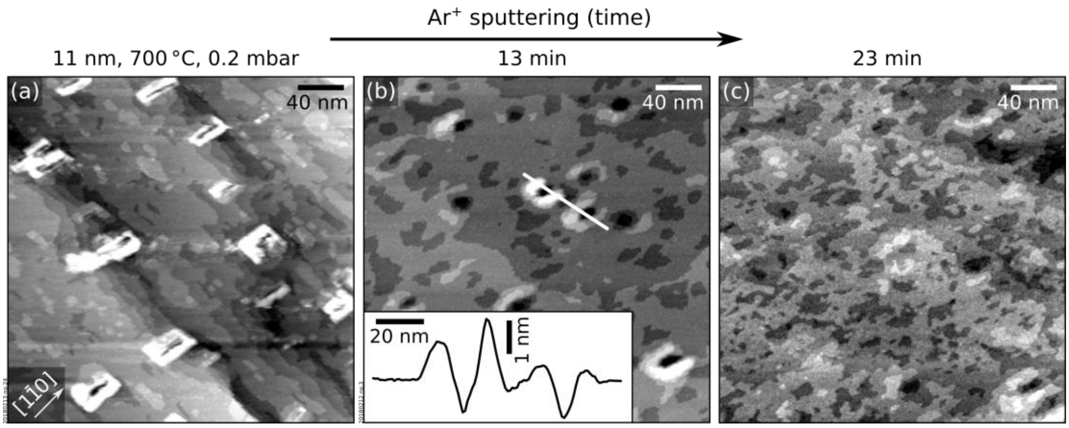

Fig. S4. Recovering ideal morphologies of an LSMO(110) film of 11 nm thickness by sputtering–annealing cycles (it is the same A-site rich film of Fig. 3c in the main text). (a–c) 300 × 300 nm^2 STM images, showing the flattening of the surface by Ar^+ sputtering followed by O_2 annealing (> 45 min, 700 °C, 0.2 mbar). (b) After 13 min sputtering (≈ 3 × 10^2 ions/nm^2), the clusters have converted into pits with a depth of a few layers, surrounded by rims a few layers high (the inset shows the profiles of two pits). (c) After a total of 23 min (≈ 5.3 × 10^2 ions/nm^2) the surface is almost atomically flat (four exposed layers).

along $[1\bar{1}0]$). This is also visible from the in-plane position of the reflexes in Fig. S2: Along $[001]$ (right column of Fig. S2), the LSMO periodicity closely matches the one of the $SrTiO_3$; along $[1\bar{1}0]$ (middle column), the LSMO reflexes are found at shorter in-plane reciprocal space positions, indicating an expansion of the LSMO lattice with respect to the substrate. The in-plane strain in the film relative to the relaxed structure has the same absolute value of ≈ 0.168% in the two directions. This corresponds to a very small deformation of the monoclinic cell, by less than 2 pm. It is tensile along $[001]$ and compressive along $[1\bar{1}0]$. In the out-of-plane $[110]$ direction, virtually no deformation is present (≈ −0.02%). It is likely that the largest relaxation occurs in correspondence of the boundary between mirror-symmetric domains. The direction of the topography modulations observed in large-area STM images fits with this interpretation.

S3. UHV-based strategies to recover ideal surface morphologies

This section discusses UHV-based treatments to improve the morphology of rough films. First, consider surfaces such as in Figs. 3a and 3c of the main text, i.e., decorated by some clusters but otherwise showing large, atomically flat, terraces. Here, Ar^+ sputtering plus high-pressure O_2 annealing can recover almost ideal surfaces. Figure S4 shows the morphology evolution of the film of Fig. S4a as a function of the sputtering time. (After each cycle, the sample was annealed for 1 h at 700 °C and 0.2 mbar O_2.) At the beginning, the surface is decorated by clusters (Fig. S4a). A closer inspection reveals that the clusters are made of square-edged rims that surround few-nanometre-deep holes. Sputtering for 13 min followed by annealing (Fig. S4b) removes most of the rims of the clusters and makes the pits more evident (see the line scan in the inset). The pits-plus-rims clusters are reminiscent of those formed during the Ti-rich homoepitaxy of $SrTiO_3$(110).[13] This supports previous findings[1] that nonstoichiometric growth causes pronounced sticking effects in both $SrTiO_3$(110) and LSMO(110). Further sputtering–annealing the surface (Fig. S4c) removes almost all the pits, yielding a flat surface with four layers exposed.

Now consider a surface like in Fig. S5a, formed by growing a film of 22 nm thickness at 1 × 10^{-2} mbar O_2 and otherwise standard deposition parameters. Mn-rich clusters dominate the film morphology. Here, Ar^+ sputtering plus high-pressure annealing does not recover a flat surface easily, see Figs. S5b–d. The number of exposed layers decreases with more cleaning cycles, but many pits remain (see Fig. S5c after 5 cycles). A significantly longer anneal (11 h) gives only a minor improvement (Fig. S5d). The most effective way to recover the surface is to anneal at more reducing conditions: Heating for 1 h at the same temperature but in UHV rather than in an O_2 background drastically reduces the number of exposed layers (Fig. S5e). A similar effect was reported for other oxide surfaces:[14] When the oxygen partial pressure (and, hence, its chemical potential) during annealing changes, reconstructions of different cation composition become more stable. The change of the surface reconstruction requires to move material across the surface to realize the thermodynamically stable composition. This helps to overcome kinetic barriers that otherwise prevent the formation of a smooth surface.

Note that sputtering always depletes the surface from Mn, shifting the surface towards the A-site rich end of LSMO(110) (see top row of Fig. 4 of the main text).[1] To recover the correct Mn stoichiometry, it is sufficient to deposit Mn plus high-pressure O_2 annealing.[1] After this step, the surface exhibits atomically flat terraces hundreds of nanometres wide (Fig. S5f).

References

1 G. Franceschi, M. Schmid, U. Diebold and M. Riva, "Atomically resolved surface phases of $La_{0.8}Sr_{0.2}MnO_3$(110) thin films", *J. Mater. Chem. A*, 2020, **8**, 22947.

2 S. Gerhold, M. Riva, B. Yildiz, M. Schmid and U. Diebold, "Adjusting island density and morphology of the $SrTiO_3$(110)-(4×1) surface: Pulsed laser deposition combined with scanning tunneling microscopy", *Surf. Sci.*, 2016, **651**, 76.

 This journal is © The Royal Society of Chemistry 2023

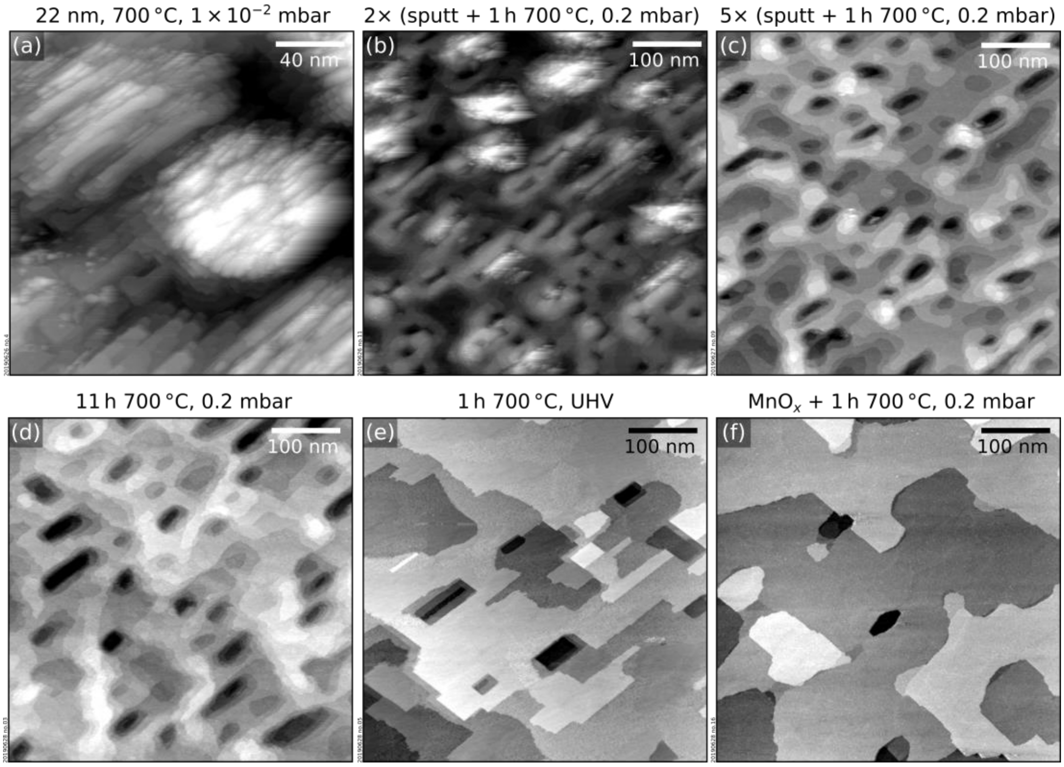

Fig. S5. Recovering the morphology of a rough, Mn-rich LSMO(110) film of 22 nm thickness grown at 1 × 10^{-2} mbar O_2 and 700 °C. STM images (a) 200 × 200 nm^2 and (b–f) and 500 × 500 nm^2 in size. Sputtering plus high-pressure O_2 annealing (1 h, 700 °C, 0.2 mbar) is partially effective in removing the excess material localized at the clusters (b, c). However, holes that are a few nanometres deep remain at the surface (c) even after 11 h annealing at the same conditions (d). With only 1 h annealing at reducing conditions (700 °C, UHV), the surface morphology improves dramatically (e). The morphology is almost ideal after depositing Mn (plus high-pressure O_2 annealing), which replenishes the surface from the Mn atoms preferentially removed by sputtering (f).

3 M. Riva, G. Franceschi, Q. Lu, M. Schmid, B. Yildiz and U. Diebold, "Pushing the detection of cation nonstoichiometry to the limit", *Phys. Rev. Mater.*, 2019, **3**, 43802.

4 D. Kriegner, E. Wintersberger and J. Stangl, "*xrayutilities*: A versatile tool for reciprocal space conversion of scattering data recorded with linear and area detectors", *J. Appl. Crystallogr.*, 2013, **46**, 1162.

5 D. S. Mebane, Y. Liu and M. Liu, "Refinement of the bulk defect model for $La_xSr_{1-x}MnO_{3\pm\delta}$", *Solid State Ionics*, 2008, **178**, 1950.

6 G. Franceschi, M. Wagner, J. Hofinger, T. Krajňák, M. Schmid, U. Diebold and M. Riva, "Growth of In_2O_3(111) thin films with optimized surfaces", *Phys. Rev. Mater.*, 2019, **3**, 1.

7 C. Dong, F. Wu and H. Chen, "Correction of zero shift in powder diffraction patterns using the reflection-pair method", *J. Appl. Crystallogr.*, 1999, **32**, 850.

8 H. D. Megaw and C. N. W. Darlington, "Geometrical and structural relations in the rhombohedral perovskites", *Acta Crystallogr. Sect. A*, 1975, **31**, 161.

9 T. Darling, A. Migliori, E. Moshopoulou, S. A. Trugman, J. Neumeier, J. Sarrao, A. Bishop and J. Thompson, "Measurement of the elastic tensor of a single crystal of $La_{0.83}Sr_{0.17}MnO_3$ and its response to magnetic fields", *Phys. Rev. B*, 1998, **57**, 5093.

10 P. M. Marcus and F. Jona, "Strains in epitaxial films: The general case", *Phys. Rev. B*, 1995, **51**, 5263.

11 P. Virtanen *et al.*, "SciPy 1.0: fundamental algorithms for scientific computing in Python", *Nat. Methods 2020*, **17**, 261.

12 S. Gražulis, D. Chateigner, R. T. Downs, A. F. T. Yokochi, M. Quirós, L. Lutterotti, E. Manakova, J. Butkus, P. Moeck and A. Le Bail, "Crystallography Open Database - An open-access collection of crystal structures", *J. Appl. Crystallogr.*, 2009, **42**, 726.

13 M. Riva, G. Franceschi, M. Schmid and U. Diebold, "Epitaxial growth of complex oxide films: Role of surface reconstructions", *Phys. Rev. Res.*, 2019, **1**, 33059.

14 G. Franceschi, M. Schmid, U. Diebold and M. Riva, "Reconstruction changes drive surface diffusion and determine the flatness of oxide surfaces", *J. Vac. Sci. Technol. A*, 2022, **40**, 023206.
